# Evolution of resistance mechanisms and biological characteristics of rifampicin-resistant *Staphylococcus aureus* strains selected in vitro

**DOI:** 10.1186/s12866-019-1573-9

**Published:** 2019-09-18

**Authors:** Chong Wang, Renchi Fang, Beibei Zhou, Xuebin Tian, Xiucai Zhang, Xiangkuo Zheng, Siqin Zhang, Guofeng Dong, Jianming Cao, Tieli Zhou

**Affiliations:** 1 Department of Clinical Laboratory, The First Affiliated Hospital of Wenzhou Medical University, Wenzhou, Zhejiang Province China; 2 School of Laboratory Medicine and Life Sciences, Wenzhou Medical University, Wenzhou, Zhejiang Province China

**Keywords:** *Staphylococcus aureus*, Rifampicin resistance, Drug resistance evolution, Fitness cost

## Abstract

**Background:**

We aimed to determine the evolutionary pathways of rifampicin resistance in *Staphylococcus aureus*, and the impact of resistance mutations in the *rpoB* gene on fitness.

**Methods:**

Three clinical strains and one reference strain were used to select for rifampicin-resistant *S. aureus* variants. The mutations responsible for rifampicin resistance in all of the selected isolates in vitro were investigated by polymerase chain reaction (PCR) and DNA sequencing. To compare the fitness cost of *rpoB* mutations against their corresponding original isolates, we performed bacterial growth curve assays, static biofilm assays, in vitro competition experiments and an infection model of *Galleria mellonella* larvae.

**Results:**

We obtained four rifampicin-resistant *S. aureus* isolates that showed high levels of resistance to rifampicin with a minimal inhibitory concentration (MIC) of 128 mg/L, and all isolates had a mutation at position 481 (H481F/Y) in RpoB. A broth microdilution assay indicated that mutation of H481F/Y did not affect susceptibility to common antibacterial drugs but slightly increased the vancomycin MIC. To identify the pathways involved in the development of rifampicin resistance, 32 variants (eight mutants for each strain) and four original isolates were selected for gene sequencing. Different generations of isolates were found to harbor various mutations sites. Compared with the corresponding original isolates, an in vitro fitness assay of the variant isolates showed that growth and virulence were reduced, with a statistically significantly decreased fitness, whereas the capacity for biofilm formation was elevated.

**Conclusions:**

Our findings suggested that the acquisition of rifampicin resistance in *S. aureus* was dynamic and was associated with a significant fitness cost.

**Electronic supplementary material:**

The online version of this article (10.1186/s12866-019-1573-9) contains supplementary material, which is available to authorized users.

## Background

*Staphylococcus aureus* is one of the major human pathogens that causes a remarkable spectrum of disease, ranging from skin infections to life-threatening endocarditis with significant morbidity and mortality in both men and women of all ages [1, 2]. The widespread increase in resistance to antimicrobials, such as vancomycin and daptomycin, and in particularly to methicillin, as a result of the misuse of antibiotics, constitutes a global challenge for the treatment of infections caused by *S. aureus* [3].

Rifampicin is an antimicrobial agent that inhibits transcription via binding to the β-subunit of the bacterial DNA-dependent RNA polymerase, which is encoded by the *rpoB* gene [4], and is used in combination therapy for serious *S. aureus* infections [5]. Unfortunately, the frequency of rifampicin resistance among *S. aureus* isolates has increased dramatically over recent times. An antimicrobial susceptibility surveillance study conducted in South Africa showed that 52.8% of multidrug-resistant *S. aureus* (MRSA) isolates from public laboratories were rifampicin-resistant during 2005 and 2006 [6], and the percentage of RIF-R MRSA isolates increased rapidly from 15.5% (in 2004) to 50.2% (in 2008) in China [7]. Rifampicin resistance is caused by mutations in a highly conserved region of the *rpoB* gene, known as the rifampicin resistance-determining region [8].

Previous studies have demonstrated that some mutations in the *rpoB* gene could alter *S. aureus* susceptibility not only to rifampicin, but also to other last-line antibiotics such as vancomycin and daptomycin [9]. Interestingly, different mutations have been found to have different impacts on pathogenesis. For example, the RIF-R mutation *rpoB-*H481Y has been associated with persistent infection via attenuated host innate immune responses [10], whereas the RIF-R mutation *rpoB-*A477D was found to confer a range of phenotypes showing extracellular matrix thickening [11]. Because mutations in the *rpoB* gene play a key role in the resistance of *S. aureus* to rifampicin, it is important to fully elucidate the effect of particular mutations on the phenotype of antibiotic resistance. Here, three clinical strains were used to select for rifampicin-resistant variants in vitro. We aimed to elucidate the pathways involved in the development of rifampicin resistance and to investigate the impact of *rpoB* mutations on phenotype, virulence and susceptibility to other antibacterial agents.

## Methods

### Bacterial isolates

Three *S. aureus* isolates (SA247, SA252, SA1370) were collected from the First Affiliated Hospital of Wenzhou Medical University in China. SA247 was isolated from sputum, and SA252 and SA1370 were isolated from wounds. All three isolates were susceptible to rifampicin (RIF-S) (minimum inhibitory concentration (MIC) = 0.25 mg/L), and were used to select for the development of rifampicin resistance. All isolates were identified by matrix-assisted laser desorption/ionization time of flight mass spectrometry (MALDI-TOF MS) using a Vitek mass spectrometer (BioMerieux, Lyons, France). In addition, *S. aureus* ATCC 25923 was used as a control strain. The strains were transferred from blood agar plates to trypsin soy broth (TSB) and were recultured for 18 h for further analysis.

### In vitro selection of RIF-R strains

An adaptation test was conducted according to the method reported previously with slight modifications [12]. Four susceptible isolates were used to select for rifampicin-resistant variants. An overnight culture of each isolate, grown in cation-adjusted Mueller–Hinton broth (CAMHB), was adjusted to an OD_600_ = 0.1, and 30 mL were inoculated into tubes containing 3 mL of MHB with graded concentrations of rifampicin: (1) 1/2 × MIC; (2) 1× MIC; (3) 2× MIC; and (4) 4 × MIC. All tubes were incubated at 37 °C overnight without shaking in the dark. The next day, the tube with visible growth at the highest rifampicin concentration was used as inoculum for the next series of tubes with increasing drug concentrations. This procedure was repeated for 15 days. The samples were passaged for 6 days in CAMHB without rifampicin and had their MICs determined again to check the stability of the phenotype.

### Antimicrobial susceptibility testing

The MICs of several antimicrobials (including cefazolin, rifampicin, vancomycin, oxacillin, clindamycin, cotrimoxazole, minocycline, linezolid, azithromycin and clarithromycin) for the wild-type strains and their corresponding resistant strains were determined by the broth microdilution method in accordance with the guidelines of the Clinical and Laboratory Standards Institute 2016 [13]. The MICs of rifampicin were interpreted according to the European Committee on Antimicrobial Susceptibility Testing 2016 breakpoints [14]. *Escherichia coli* ATCC 25922 and *S. aureus* ATCC 29213 were used as control strains.

### PCR detection and sequencing of resistance genes

The *rpoB* gene was amplified by PCR using the primers *rpob*SP1F-5′-TTATGCTGCACCTTCGTG-3′ and *rpob*SP5R-5′-CAAGTGCCCATACCTCCCATC-3′, designed to amplify the entire region of interest. The genomic DNAs of the isolates were extracted using the Biospin Bacterial Genomic DNA Extraction kit (Bioflux, Tokyo, Japan) and were employed as the templates for PCR amplification. The PCR products were sequenced by Shanghai Genomics Institute Technology Co. Ltd. (Shanghai, China). The obtained nucleotide sequences were analyzed and compared by BLAST searches against the NCBI database (www.ncbi.nl m.nih.gov/BLAST).

### Bacterial growth curve with kinetic parameters

The *S. aureus* isolates were cultured in TSB at 37 °C for 24 h to obtain bacterial growth curves. Overnight culture was then transferred to fresh TSB at a ratio of 1 to 100 and incubated at 37 °C without agitation. The optical density of the bacterial culture at 595 nm (OD_595_) was measured after 0 to 24 h of incubation. Experiments were performed in duplicate, and the averages were used for estimating growth parameters.

### Static biofilm assays

Biofilm determination was conducted according to a previous report with minor modifications [15]. *S. aureus* isolates were incubated statically overnight at room temperature in 96-well plates containing TSB and were subsequently diluted 1:50 in 100 mL of TSB in duplicate 96-well plates (Falcon; BD Biosciences, San Jose, CA, USA). Following 24 h incubation at 37 °C, planktonic bacteria were decanted and the wells were washed twice with distilled water. Biofilms attached to the well surfaces were stained for 15 mins at room temperature with 125 mL of 0.1% (w/v) Crystal Violet solution (Sigma-Aldrich). The Crystal Violet solution was decanted and wells were subsequently washed twice with distilled water. The bound dye was solubilized from adherent cells with 33% acetic acid and subsequently quantified by measuring the absorbance at 595 nm. The data for each strain represented average values taken from four replicate wells performed in two independent experiments.

### In vitro competition experiments

Experiments were performed to measure the in vitro competition between resistant and susceptible strains. Exponentially growing cells of the corresponding and strain were mixed in a 1:1 proportion and resuspended in 0.9% saline solution. Approximately 10^3^ cells from each mixture were inoculated into 10-ml flasks of LB broth and grown at 37 °C and 180 rpm for 16 to 18 h, which corresponds to approximately 20 cell generations. Serial 10-fold dilutions were plated in duplicate onto LB agar (LBA) without drug and LBA with 32 μg/mL of rifampicin in order to determine, respectively, the total number of CFU and the CFU of the rifampicin-resistant strains, after overnight incubation at 37 °C. The competition index (CI) was defined as the ratio between the CFU of the rifampicin-resistant strain and the rifampicin-susceptible strain. The CI values were calculated for each independent competition assay, and the median values were calculated too.

### Infection model of *Galleria mellonella* larvae

*G. mellonella* killing assays were carried out as described previously with slight modifications [16]. *G. mellonella* was used as an in vivo infection model to compare the virulence differences between resistant and susceptible strains. Twelve caterpillars weighing between 200 and 250 mg were randomly selected for each isolate. Larvae were injected with 10 μL of bacterial suspension containing 5 × 10^8^ CFU/mL dilutions in phosphate-buffered saline (PBS), into the last left proleg using a 25 μL Hamilton precision syringe. Uninfected larvae (12 untreated or injected with 10 μL PBS) were used as a control. The insects were incubated at 37 °C in the dark and were observed after 24, 48 and 72 h. Insects were considered dead when they repeatedly failed to respond to physical stimuli. The primary outcome for the insect model was rapidity and extent of mortality of *G. mellonella*, as assessed by Kaplan-Meier analysis.

### Statistical analysis

All statistical data were calculated by SPSS 17.0 (SPSS Inc., IL, USA). For the colony counting assay, the unpaired Student’s *t*-test (two-tailed) was used. Calculated *P* values of < 0.05 were considered to indicate statistical significance.

## Results

### In vitro selection of RIF-R isolates

To identify pathways involved in the development of rifampicin resistance, three rifampicin-susceptible isolates and *S. aureus* ATCC 25923 were exposed to graded levels of the drug, with a starting concentration of 0.125 mg/L. After eight generations of selection and passaging for 6 days in MHB without rifampicin, a total of 32 variant strains (eight from each isolate) from the four original strains were obtained: SA247 (1st–8th); SA252 (1st–8th); SA1370 (1st–8th); SA25923 (1st–8th) (the number represents the corresponding generation). All of the eighth generation (8th) strains that had been passaged for 6 days expressed high level rifampicin resistance (SA247R, SA252R, SA370R, SA25923R; rifampicin MIC = 128 mg/L).

### Antimicrobial susceptibility patterns

Antimicrobial susceptibility testing by the broth microdilution method revealed that three original strains were susceptible to all of the tested antimicrobials, except penicillin, in this study. The sensitivity of the 8th generation strains were consistent with the corresponding original strains, with the exception of rifampicin. The resistance phenotypes determined for the *S. aureus* isolates are shown in Table 1.

**Table 1:** MICs of the antibiotics tested in the current study

Strains	MIC (mg / L)										
	RIF	VAN	TEC	GEN	LNZ	TCY	ERY	CIP	OXA	CLI	CZO
SA247	0.25	0.25	0.25	0.5	2	0.25	0.25	0.25	0.5	0.25	0.25
SA247R	128	0.5	1	0.5	2	0.25	0.25	0.25	0.5	0.25	0.25
SA252	0.25	0.5	0.25	0.5	2	0.25	0.25	0.5	0.25	0.25	0.5
SA252R	128	1	1	0.5	2	0.25	0.25	0.5	0.25	0.25	0.5
SA1370	0.25	0.25	0.5	0.5	1	0.25	64	4	0.25	4	0.25
SA1370R	128	1	1	0.5	1	0.25	64	4	0.25	4	0.25
ATCC 25923	0.25	0.25	0.5	0.5	1	0.25	0.25	0.5	0.25	0.25	0.25
ATCC25923R	128	0.5	1	0.5	1	0.25	0.25	0.5	0.25	0.25	0.25

*MICs* minimum inhibitory concentrations; *RIF* rifampicin; *VAN* vancomycin; *TEC* teicoplanin; *GEN* gentamicin; *LNZ* linezolid; *TCY* tetracycline; *ERY* erythromycin; *CIP* ciprofloxacin; *OXA* oxacillin; *CLI* clindamycin; *CZO* cefazolin

### Distribution of mutations associated with rifampicin resistance

To identify changes that correlated with rifampicin resistance acquisition, all of the strains were selected for gene sequencing. All four independent experiments followed the same evolutionary trajectory in achieving rifampicin resistance, and the results are shown in Table 2. Via sequencing analysis, no amino acid substitutions were observed in the RpoB protein of the 1st generation strains; however, the specific amino acid mutated varied among the 2nd to 8th generation strains. Within 6 days of passage, all 8th generation strains harbored at least a single amino acid substitution in the protein encoded by the *rpoB* gene.

**Table 2:** MIC and mutation of each generation of strains

Strains	Generations									
		0th	1th	2th	3th	4th	5th	6th	7th	8th
SA247R	MIC	0.25	0.25	1	2	4	4	8	32	128
	Mutation	–	–	–	H481L	H481L	H481L	H481L	H481F	H481F
SA252R	MIC	0.25	0.25	0.5	2	4	8	16	64	128
	Mutation	–	–	–	–	–	–	H481Y	H481Y	H481Y
SA1370R	MIC	0.25	0.25	0.5	1	2	8	16	32	128
	Mutation	–	–	–	–	–	–	S464P	H481Y/S464P	H481Y/S464P
ATCC 25923R	MIC	0.25	0.25	1	2	4	16	32	64	128
	Mutation	–	–	–	R484H	S464P	S464P	S464P	H481Y/S464P	H481Y/S464P

*MIC* minimum inhibitory concentration, *mg/L*; *H* Histidine; *L* Leucine; *F* Phenylalanine; *Y* Tyrosine; *S* Serine; *P* Proline; *R* Arginine

### Resistance to rifampicin associated with a fitness cost in *S. aureus*

**Fig. 1:**
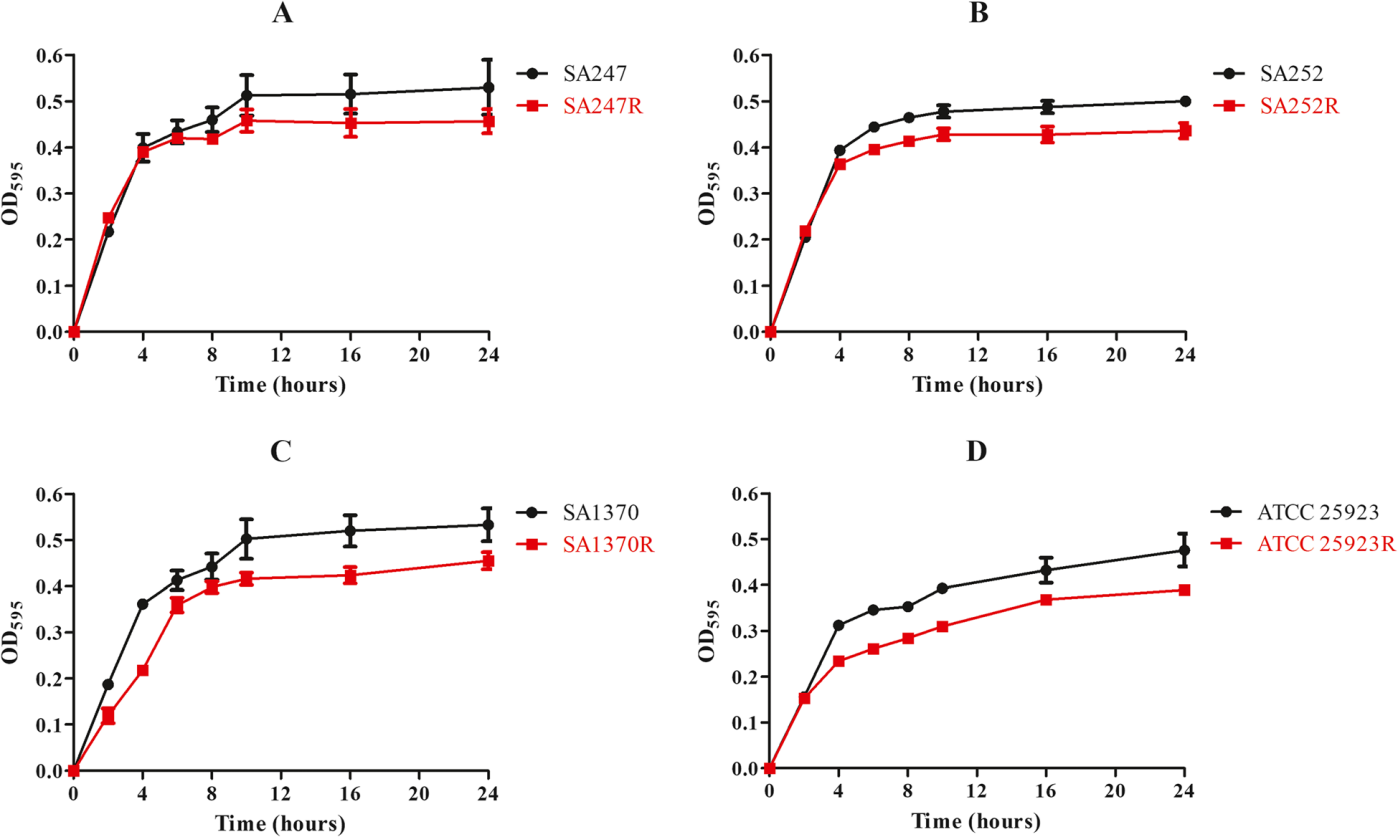
Bacterial growth curves for all strains derived in this study. (**a)** Growth curves for SA247 (original strain) and SA247R (8th generation strain); (**b**) growth curves for SA252 and SA252R; (**c**) growth curves for SA1370 and SA1370R; (**d**) growth curves for ATCC 25923 and ATCC 25923R

**Fig. 2:**
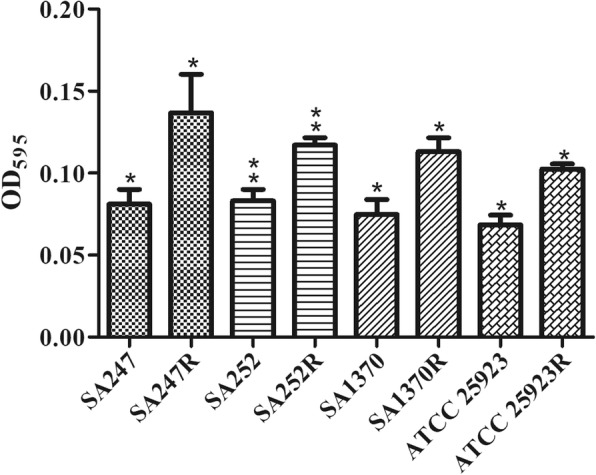
The biofilm formation ability of all strains derived in this study

**Fig. 3:**
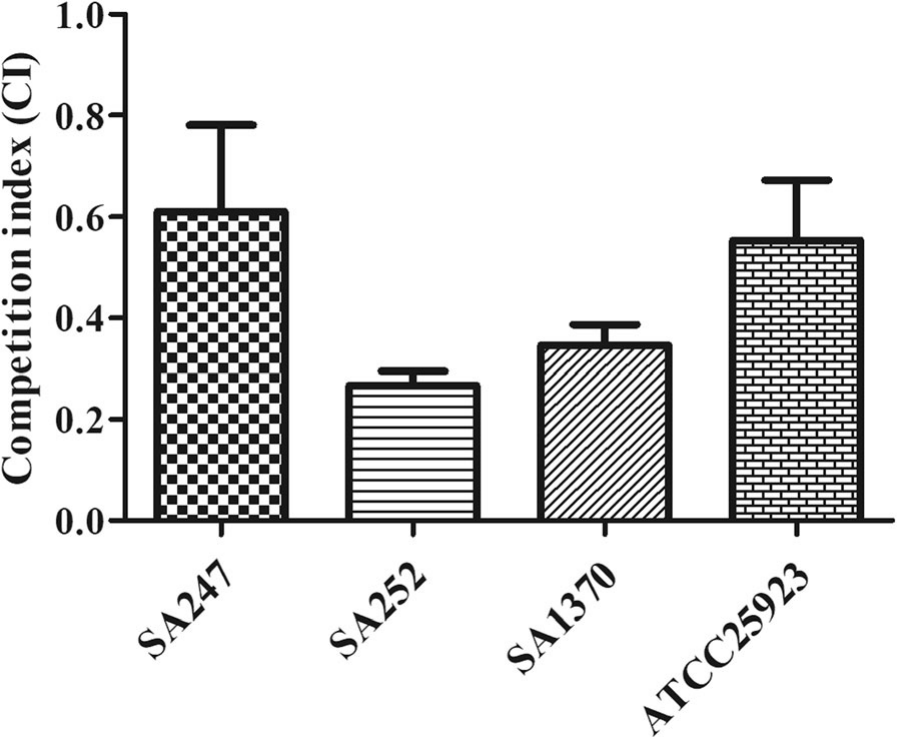
In vitro competition index (CI) results of all strains derived in study

To assess whether the evolution of rifampicin resistance strains was accompanied by an associated fitness cost, we measured bacterial growth curves and analyzed biofilm formation ability for all strains derived from this study. Growth curves revealed that the growth rates were faster for the original strains than for the corresponding 8th generation strains (Fig. 1 and Additional file 1). The biofilm-forming ability was variable among strains from different patients. When compared with the corresponding original strains, the biofilm formation ability of the resistant strains was changed (Fig. 2 and Additional file 2). In vitro competition experiments, we observed a marked decrease in fitness for these strains, as shown by the competition index (CI) results in Fig. 3 and Additional file 3. The greatest decrease was observed for the SA252 strain, with a median CI of 0.26, followed by the SA1370, ATCC25923 and SA247 isolates, with median CIs of 0.34, o.55 and 0.61, respectively.

### Infection model of *Galleria mellonella* larvae

**Fig. 4:**
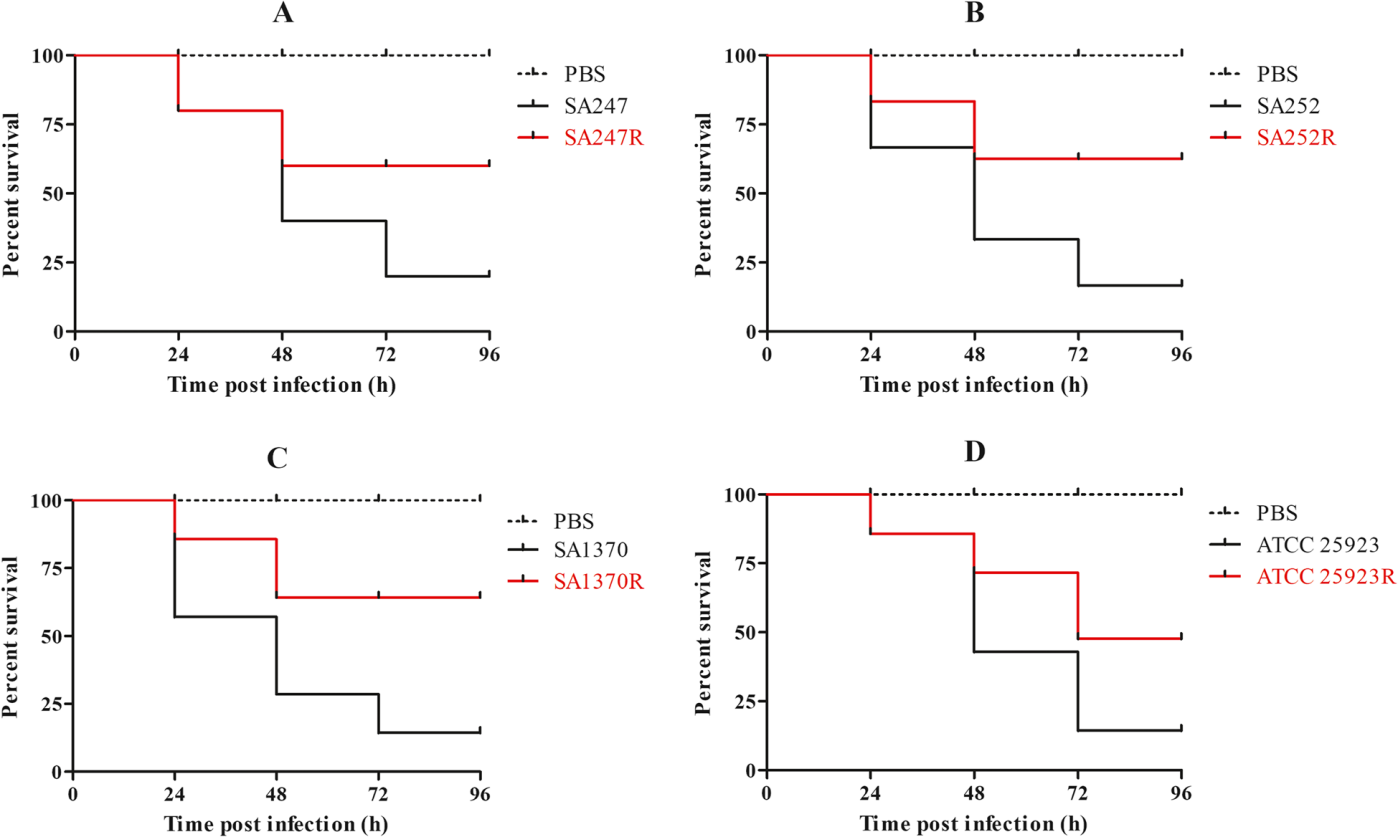
Infection model of *Galleria mellonella* larvae. (**a**) Survival curves of *Galleria mellonella* larvae infected with S247 and S247R; (**b**) Survival curves of Galleria mellonella larvae infected with S252 and S252R; (**c**) Survival curves of Galleria mellonella larvae infected with S1370 and S1370R; (**d**) Survival curves of Galleria mellonella larvae infected with ATCC 25923 and ATCC 25923R

To determine the virulence of strains with rifampicin resistant and sensitive phenotypes, larvae were injected with 5 × 10^8^ CFU of bacterial suspension and their survival was monitored (Fig. 4 and Additional file 4). At 96 h post-infection, the mortality of larvae was lower for RIF-R *S. aureus* isolates compared with the corresponding RIF-S strains. No mortality was observed in the control injected with PBS.

## Discussion

Despite effective antibiotics and constant improvements in patient care, *S. aureus* infections are still common in both hospitals and the community. Unfortunately, early intervention with appropriate antimicrobial therapies, such as daptomycin and vancomycin, is becoming less effective [10]. Therefore, the treatment of *S. aureus* infections with combination therapy remains an area of active research interest. Rifampicin shows activity against *S. aureus* and is one of the drugs used in combination therapy [5]. However, increasing rifampicin resistance caused by mutations in the *rpoB* gene encoding RNA polymerase has been detected, challenging the use of recommended treatment regimens [17]. The molecular mechanisms underlying rifampicin resistance in *S. aureus* were investigated. Many previous studies have demonstrated that mutations in a conserved region of the *rpoB* gene, known as the rifampicin resistance-determining region, are responsible for rifampicin resistance, but little has been reported regarding the pathways involved in the development of rifampicin resistance. The purpose of this study was to determine the *rpoB* gene mutations that confer RIF-R in *S. aureus* strains during the process of in vitro selection and the phenotypic consequences linked to rifampicin resistance.

In the present study, we obtained four RIF-R *S. aureus* strains with rifampicin MICs > 128 mg/L via in vitro selection. As one of the drugs used in combination therapy, rifampicin resistance compromises susceptibility to other last-line antibiotics such as vancomycin and daptomycin. Drug susceptibility screening of *S. aureus* showed that only rifampicin-resistant strains differed significantly from the original strains. Some reports have suggested that the rate of rifampicin resistance is clinically significant when used in monotherapy, this may be because the evolution of antimicrobial resistance is increased compared with combination therapy [18]. With rifampicin resistance being so easily acquired, it seems inappropriate to consider rifampicin monotherapy for the treatment of *S. aureus* infection. However, research has shown that certain mutations in the *rpoB* gene are associated with alterations in vancomycin susceptibility in *S. aureus*. Here, we amplified and sequenced portions of the *rpoB* gene from RIF-R *S. aureus* strains, and a H481Y point mutation was the only substitution detected among them. As described in the literature, position 481 of RpoB presents a hot-spot for amino acid residue replacement and has recently been found to be the strongest genetic marker of increased vancomycin resistance [19, 20]. A previous study confirmed that H481Y is associated with decreased susceptibility to vancomycin by genetic reconstruction [21]. Of interest is the observation that all strains were susceptible to vancomycin (MIC = 1 mg/L), suggested that this mutational change was not associated with resistance to vancomycin.

Previous studies have shown that high level rifampicin resistance may be attributed to multiple mutations, indicating a step-by-step mechanism in resistance development [22]. Therefore, dynamic changes in the rifampicin resistance of *S. aureus* strains are of urgent concern. All of the strains obtained in our experiments were subjected to genome sequencing, to identify changes that correlated with the acquisition of rifampicin resistance. Sequencing revealed that the mutations were dynamic and remained stable until high levels of resistance were achieved. These mutations may indicate genetic drift or may reflect the selective pressure imposed by rifampicin for a strain to develop resistance.

Notably, certain rifampicin alleles often lead to adapted strains having different physiological characteristics compared with their original strains. To verify these physiological characteristics, we conducted in vitro adaptability tests, including growth curve analysis, biofilm formation ability assays, in vitro competition experiments and an infection model of *G. mellonella* larvae. To determine whether rifampicin resistance acquired in vitro may impact virulence traits in *S. aureus*, *G. mellonella* was used as an in vivo model. Worms injected with PBS showed 100% survival at day 4, but those injected with the original strains displayed low survival rates. A similar trend was observed with strain ATCC 25923, although the survival rate was higher than with the original *S. aureus* strain*.* The growth curves of each resistant strain were lower than their original strains, suggesting that RIF-R is associated with a reduction in the virulence fitness cost. However, these mutations in the *rpoB* gene could induce biofilm formation. The ability to form a biofilm is an important virulence factor, and we have previously reported that biofilm production is a sort of defense reaction of *S. aureus* [23]*.* The outcome of the competition process depends on the relative fitness, defined as the efficacy of multiplication of the resistant cell compared with that of the susceptible cell. And, the data showed that the median CI values for these strains were associated with a statistically significantly decreased fitness in the in vitro experiments. Based on the above results*,* we hypothesize that when selecting for RIF-R *S. aureus* clones carrying specific *rpoB* mutations conferring a lower fitness cost and adaptive positive pleiotropic effects, stable conversion to rifampin-resistant lineages can occur.

## Conclusions

In conclusion, we found that rifampicin resistance was closely associated with *rpoB* gene mutations. Our results also showed that mutations in the *rpoB* gene were not associated with decreased susceptibility to vancomycin in *S. aureus*. It is worth noting that rifampicin is the major selective pressure driving *rpoB* evolution, and plays a key role in enhancing pathogenicity. Therefore, rational use of antibiotics is important to prevent the emergence of resistant bacteria.

## Additional files

Additional file 1: **Table S1**. The details of bacterial growth curves for all strains derived in this study, the optical density of the bacterial culture at 595 nm was measured after 0 to 24 h of incubation. Experiments were performed in duplicate, and the averages were used for estimating growth parameters. (DOCX 41 kb)

Additional file 2: **Table S2**. The details of biofilm formation ability of all strains derived in this study, quantified by measuring the absorbance at 595 nm, and the data for each strain represented average values taken from four replicate wells performed in two independent experiments. (DOCX 20 kb)

Additional file 3: **Table S3**. The details of in vitro competition index (CI) results of all strains derived in this study, CI was defined as the ratio between the CFU of the rifampicin-resistant strain and the rifampicin-susceptible strain. (DOCX 18 kb)

Additional file 4: **Table S4**. The details of infection model of *Galleria mellonella* larvae, larvae were injected with 5 × 108 CFU of bacterial suspension and their survival was monitored, at 96 h post-infection, the mortality of larvae was lower for RIF-R *S. aureus* isolates compared with the corresponding RIF-S strains. (DOCX 17 kb)

## Data Availability

All data generated or analyzed during this study are included in this published article.

## Abbreviations

MALDI-TOF MS: Matrix-assisted laser desorption/ionization time of flight mass spectrometry
MIC: Minimum inhibitory concentration
PCR: Polymerase chain reaction
