# Supplementary material for: Evolution of resistance mechanisms and biological characteristics of rifampicin-resistant Staphylococcus aureus strains selected in vitro
Source: BMC Microbiol. 2019 Sep 18;19:220. doi: 10.1186/s12866-019-1573-9 (PMC6751903; doi:10.1186/s12866-019-1573-9)
Supplement: Supplementary file 1 — Table S1. The details of bacterial growth curves for all strains derived in this study, the optical density of the bacterial culture at 595 nm was measured after 0 to 24 h of incubation. Experiments were performed in duplicate, and the averages were used for estimating growth parameters. (DOCX 41 kb) [file 12866_2019_1573_MOESM1_ESM.docx]

**Table S1. Bacterial growth curves for all strains derived in this study.**

| **Times(h)** | **SA247** | **SA247** | **SA247** | **SA247R** | **SA247R** | **SA247R** |
| --- | --- | --- | --- | --- | --- | --- |
| 0 | 0.000 | 0.000 | 0.000 | 0.000 | 0.000 | 0.000 |
| 2 | 0.225 | 0.222 | 0.204 | 0.255 | 0.245 | 0.242 |
| 4 | 0.429 | 0.400 | 0.369 | 0.394 | 0.389 | 0.388 |
| 6 | 0.459 | 0.433 | 0.409 | 0.425 | 0.419 | 0.417 |
| 8 | 0.490 | 0.452 | 0.438 | 0.430 | 0.415 | 0.410 |
| 10 | 0.557 | 0.511 | 0.470 | 0.486 | 0.446 | 0.442 |
| 16 | 0.560 | 0.511 | 0.476 | 0.488 | 0.435 | 0.436 |
| 24 | 0.596 | 0.513 | 0.481 | 0.487 | 0.443 | 0.441 |

| **Times(h)** | **SA252** | **SA252** | **SA252** | **SA252R** | **SA252R** | **SA252R** |
| --- | --- | --- | --- | --- | --- | --- |
| 0 | 0.000 | 0.000 | 0.000 | 0.000 | 0.000 | 0.000 |
| 2 | 0.211 | 0.207 | 0.196 | 0.219 | 0.222 | 0.215 |
| 4 | 0.395 | 0.393 | 0.393 | 0.367 | 0.361 | 0.363 |
| 6 | 0.444 | 0.441 | 0.448 | 0.395 | 0.405 | 0.388 |
| 8 | 0.460 | 0.470 | 0.464 | 0.415 | 0.422 | 0.404 |
| 10 | 0.464 | 0.490 | 0.480 | 0.423 | 0.443 | 0.419 |
| 16 | 0.473 | 0.499 | 0.491 | 0.430 | 0.444 | 0.410 |
| 24 | 0.496 | 0.509 | 0.496 | 0.438 | 0.452 | 0.419 |

| **Times(h)** | **SA1370** | **SA1370** | **SA1370** | **SA1370R** | **SA1370R** | **SA1370R** |
| --- | --- | --- | --- | --- | --- | --- |
| 0 | 0.000 | 0.000 | 0.000 | 0.000 | 0.000 | 0.000 |
| 2 | 0.191 | 0.184 | 0.185 | 0.111 | 0.108 | 0.137 |
| 4 | 0.365 | 0.350 | 0.369 | 0.220 | 0.209 | 0.224 |
| 6 | 0.406 | 0.396 | 0.436 | 0.370 | 0.341 | 0.366 |
| 8 | 0.440 | 0.415 | 0.471 | 0.411 | 0.386 | 0.396 |
| 10 | 0.480 | 0.475 | 0.551 | 0.430 | 0.403 | 0.414 |
| 16 | 0.498 | 0.502 | 0.559 | 0.440 | 0.405 | 0.425 |
| 24 | 0.506 | 0.519 | 0.573 | 0.453 | 0.437 | 0.474 |

| **Times(h)** | **ATCC 25923** | **ATCC 25923** | **ATCC 25923** | **ATCC 25923R** | **ATCC 25923R** | **ATCC 25923R** |
| --- | --- | --- | --- | --- | --- | --- |
| 0 | 0.000 | 0.000 | 0.000 | 0.000 | 0.000 | 0.000 |
| 2 | 0.159 | 0.153 | 0.155 | 0.153 | 0.153 | 0.151 |
| 4 | 0.310 | 0.305 | 0.322 | 0.233 | 0.232 | 0.236 |
| 6 | 0.346 | 0.343 | 0.348 | 0.259 | 0.259 | 0.264 |
| 8 | 0.340 | 0.358 | 0.360 | 0.285 | 0.281 | 0.286 |
| 10 | 0.399 | 0.391 | 0.389 | 0.311 | 0.308 | 0.311 |
| 16 | 0.401 | 0.452 | 0.443 | 0.380 | 0.357 | 0.368 |
| 24 | 0.435 | 0.497 | 0.496 | 0.390 | 0.388 | 0.390 |
