# Supplementary material for: Evolution of resistance mechanisms and biological characteristics of rifampicin-resistant Staphylococcus aureus strains selected in vitro
Source: BMC Microbiol. 2019 Sep 18;19:220. doi: 10.1186/s12866-019-1573-9 (PMC6751903; doi:10.1186/s12866-019-1573-9)
Supplement: Supplementary file 2 — Table S2. The details of biofilm formation ability of all strains derived in this study, quantified by measuring the absorbance at 595 nm, and the data for each strain represented average values taken from four replicate wells performed in two independent experiments. (DOCX 20 kb) [file 12866_2019_1573_MOESM2_ESM.docx]

**Table S2. The biofilm formation ability of all strains derived in this study.**

| **SA247** | **SA247R** | **SA252** | **SA252R** | **SA1370** | **SA1370R** | **ATCC 25923** | **ATCC 25923R** |
| --- | --- | --- | --- | --- | --- | --- | --- |
| 0.091 | 0.161 | 0.080 | 0.118 | 0.064 | 0.112 | 0.074 | 0.100 |
| 0.079 | 0.114 | 0.078 | 0.112 | 0.079 | 0.105 | 0.062 | 0.101 |
| 0.073 | 0.135 | 0.091 | 0.121 | 0.081 | 0.122 | 0.069 | 0.106 |
