# Supplementary material for: Evolution of resistance mechanisms and biological characteristics of rifampicin-resistant Staphylococcus aureus strains selected in vitro
Source: BMC Microbiol. 2019 Sep 18;19:220. doi: 10.1186/s12866-019-1573-9 (PMC6751903; doi:10.1186/s12866-019-1573-9)
Supplement: Supplementary file 3 — Table S3. The details of in vitro competition index (CI) results of all strains derived in this study, CI was defined as the ratio between the CFU of the rifampicin-resistant strain and the rifampicin-susceptible strain. (DOCX 18 kb) [file 12866_2019_1573_MOESM3_ESM.docx]

**Table S3. In vitro competition index (CI) results of all strains derived in this study.**

| **Strains** | **SA247** | **SA252** | **SA1370** | **ATCC25923** |
| --- | --- | --- | --- | --- |
| CI | 0.79 | 0.25 | 0.31 | 0.50 |
|  | 0.45 | 0.25 | 0.34 | 0.47 |
|  | 0.59 | 0.30 | 0.39 | 0.69 |
