# Supplementary material for: Evolution of resistance mechanisms and biological characteristics of rifampicin-resistant Staphylococcus aureus strains selected in vitro
Source: BMC Microbiol. 2019 Sep 18;19:220. doi: 10.1186/s12866-019-1573-9 (PMC6751903; doi:10.1186/s12866-019-1573-9)
Supplement: Supplementary file 4 — Table S4. The details of infection model of Galleria mellonella larvae, larvae were injected with 5 × 10^8^ CFU of bacterial suspension and their survival was monitored, at 96 h post-infection, the mortality of larvae was lower for RIF-R S. aureus isolates compared with the corresponding RIF-S strains. (DOCX 17 kb) [file 12866_2019_1573_MOESM4_ESM.docx]

**Table S4. Infection model of *Galleria mellonella* larvae.**

| **Hours** | **Survivals** | | |
| --- | --- | --- | --- |
|  | **PBS** | **SA247** | **SA247R** |
| **0.** | **12** | **12** | **12** |
| **24.** | **12** | **11** | **11** |
| **48.** | **12** | **9** | **10** |
| **72.** | **12** | **8** | **10** |
| **96.** | **12** | **8** | **10** |

| **Hours** | **Survivals** | | |
| --- | --- | --- | --- |
|  | **PBS** | **SA252** | **SA252R** |
| **0.** | **12** | **12** | **12** |
| **24.** | **12** | **10** | **11** |
| **48.** | **12** | **8** | **10** |
| **72.** | **12** | **7** | **10** |
| **96.** | **12** | **7** | **10** |

| **Hours** | **Survivals** | | |
| --- | --- | --- | --- |
|  | **PBS** | **ATCC 25923** | **ATCC 25923R** |
| **0.** | **12** | **12** | **12** |
| **24.** | **12** | **11** | **11** |
| **48.** | **12** | **8** | **10** |
| **72.** | **12** | **6** | **9** |
| **96.** | **12** | **6** | **9** |

| **Hours** | **Survivals** | | |
| --- | --- | --- | --- |
|  | **PBS** | **SA1370** | **SA1370R** |
| **0.** | **12** | **12** | **12** |
| **24.** | **12** | **9** | **11** |
| **48.** | **12** | **7** | **10** |
| **72.** | **12** | **6** | **10** |
| **96.** | **12** | **6** | **10** |
